# Anxiety is not the right choice! Individual differences in trait anxiety modulate biases in pseudoneglect

**DOI:** 10.3389/fnhum.2023.1201898

**Published:** 2023-08-02

**Authors:** Stefania Righi, Viola Benedetti, Fiorenza Giganti, Maria Teresa Turano, Greta Raduazzo, Maria Pia Viggiano

**Affiliations:** ^1^Department of Neurofarba, University of Florence, Florence, Italy; ^2^Fondazione Turano Onlus, Rome, Italy

**Keywords:** trait anxiety, pseudoneglect, emotion, visual attention, hemispheric asymmetry

## Abstract

Pseudoneglect, the tendency to display a leftward perceptual bias, is consistently observed in line bisection tasks. Some studies have shown that pseudoneglect is sensitive to emotions. This emotion-related modulation is likely related to valence-dependent hemispheric lateralization, although the results do not converge. A possible explanation for these inconsistencies could be individual differences in emotional tone. Considering that negative and positive emotions produce different basic activations of the two hemispheres, emotional characteristics of the subjects, such as trait anxiety, could in fact modulate the pseudoneglect phenomenon. To verify this, high- and low-anxiety participants were asked to centrally bisect horizontal lines delimited by neutral or emotional (happy and sad) faces. In line with previous studies, results here showed a decrease in the leftward bisection error in the presence of happy faces, indicating a greater involvement of the left hemisphere in processing positive emotional stimuli. In addition, trait anxiety influenced the magnitude of the visual bias. High-anxiety subjects, compared to low-anxiety subjects, showed a general bias in visual attention toward the left space as a function of emotional valence. Results are discussed within the framework of valence-dependent hemispheric specialization and the relative degree of activation. In sum, our data highlight the relevance of considering emotional individual differences in studying the pseudoneglect phenomenon.

## 1. Introduction

The myriad of stimuli that daily affect our senses are explored asymmetrically by attention in searching for and selecting relevant information to react adequately to environmental challenges (Phelps et al., 2006; Turano et al., 2017). Asymmetry in spatial exploration leads most people to start their visual search with left-sided items and to bisect horizontal lines faintly to the left of the center line (Azouvi et al., 2006; Strappini et al., 2021), depending also on the nature of the stimulus and their handedness (Viggiano and Vannucci, 2002). This leftward attentional bias is called pseudoneglect, and it is typically assessed using different tasks, such as the line bisection task, landmark task, grayscales task, tactile rod bisection task, and lateralized visual detection task (for a review, see Friederich et al., 2018). Among the tasks employed to evaluate pseudoneglect, the visual line bisection task is the most commonly used (Friederich et al., 2018). In this task, participants are required to bisect a horizontal line drawn on paper or presented by a computer. This task is a sensitive test to investigate attentional and motor biases in both healthy and brain-damaged individuals. However, studies using bisection tasks show that several factors such as age, gender, performing hand, and the direction in which participants initiate motor scanning can modulate pseudoneglect (for a review, see Jewell and McCourt, 2000). Moreover, several studies showed that higher-level cognitive processes can also influence bisection performance, mainly when the stimulus attributes have been manipulated (Fischer, 2001). For example, Ranzini et al. (2011) showed in healthy subjects that flanking a line with images of hands representing different actions influences their performance in the direction of the action that is more compatible with the object. Using numerical and non-numerical flankers and varying levels of luminance, Ranzini and Girelli (2012) observed a reciprocal interference in performance when these dimensions are combined together. In addition, Bonato et al. (2008), using directional (eye gaze) and numerical cues in a line bisection task, observed that the performance of neglect patients was influenced by task-irrelevant cues.

It is likely that the pseudoneglect phenomenon depends on the asymmetrical brain organization of visuospatial attention, which is controlled by right frontoparietal networks (Corbetta and Shulman, 2011), although the precise underlying mechanisms are not yet clear. Some theories (activation-orientation theories) attributed the pseudoneglect phenomenon to a right hemispheric dominance that would determine an attentional bias toward the contralateral hemifield (Nicholls and Roberts, 2002; Bultitude and Davies, 2006). Another hypothesis (Jewell and McCourt, 2000) suggested that the magnitude of pseudoneglect may indeed depend on the interaction of many previously mentioned variables, such as gender, age, performing hand, and the direction in which participants initiate motor scanning. Recently, this phenomenon has also been observed as a function of emotion, a variable that might modulate its magnitude. A recent review (Strappini et al., 2021) has pointed out the importance of investigating the role of emotion in modulating attentional bias, which is mediated by lateralized cerebral networks.

From a theoretical point of view, several theories considering hemispheric asymmetries in selective spatial attention have highlighted the crucial role played by emotional processing, but how and to what extent the two hemispheres superintend the influence of emotion on spatial attention remains a topic of debate. Proponents of the “right hemisphere dominance model” suggest that all emotions are processed by the right hemisphere (Borod et al., 1998, 2002; Adolphs et al., 2001; Mandal and Ambady, 2004; Gainotti, 2005). Whereas those who support the “valence-specific hypothesis” postulate the main role of the left hemisphere for positive emotions (e.g., happiness), while negative ones (e.g., anger, sadness, disgust, and fear) involve the right hemisphere (Reuter-Lorenz et al., 1983; Davidson, 1992, 1998).

A third model was proposed by Davidson (1992, 1998): the “approach–withdrawal” hypothesis. This model posits that the brain asymmetries observed for emotions are related to two distinct underlying motivational systems that orient our behaviors. The “approach system” (left side of the brain) would be typically activated by emotions such as happiness and anger because both drive the subject to approach the object that elicited them (the happiness to enjoy the object or the anger to attack the object). Differently, the “withdrawal system” (right side of the brain) would be related to sadness, fear, and disgust, all emotions that produce avoidance of the object that stimulated them.

Increased activation of the left frontal cortex (Schepman et al., 2012) is associated with an approach-related behavior that is typically displayed in the context of moving toward a desired goal. Conversely, activation of the right frontal area is related to withdrawal-related behavior that is exhibited by moving away, voluntarily or automatically, from or avoiding threatening stimuli (Dolan, 2002; Adolphs and Spezio, 2006). Based upon these assumptions, spatial attention tasks such as the line bisection task have been coupled with emotional stimuli with the aim of assessing the modulatory effect of emotion on pseudoneglect to better understand brain asymmetries in emotional processing. In fact, if emotion processing is always right-lateralized (i.e., “right-hemisphere hypothesis,” Borod et al., 1998, 2002; Adolphs et al., 2001; Mandal and Ambady, 2004; Gainotti, 2005), we can predict enhancement in pseudoneglect (leftward bias) when emotional faces flank the line to bisect. Differently, if positive emotion is left-lateralized and negative emotion is right-lateralized (i.e., the “valence-specific hypothesis,” Davidson, 1992, 1998), only negative emotions should produce an enhancement in pseudoneglect due to greater activation of the right hemisphere. Otherwise, positive emotions should reduce or eliminate pseudoneglect because of enhanced brain activity in the left hemisphere.

Considering the “approach–withdrawal” hypothesis (Davidson, 1992, 1998), positive emotions such as happiness but also negative emotions such as anger should reduce pseudoneglect phenomena because both of these emotions activate approaching behaviors that are related to greater activity of the left hemispheres. Differently, negative emotions such as fear, disgust, and sadness, which prompt an avoidance behavior, should activate more the right hemisphere and hence enhance the pseudoneglect.

However, it is still unclear whether and to what extent the emotional valence of stimuli actually influences pseudoneglect because only a few studies have found an emotion-related modulation in the allocation of visual attention (Vuilleumier and Schwartz, 2001; Pessoa, 2010). In this regard, it has been observed that, in patients with pathological neglect, the happy and angry faces were both more effective than neutral faces in reducing rightward bisection bias (Tamietto et al., 2005). However, the few studies that explored in healthy controls the effect of emotional faces on the pseudoneglect phenomenon produced conflicting results (see, for a review, Strappini et al., 2021). To the best of our knowledge, only a few studies have specifically investigated how the presentation of emotional faces with positive and negative valence modulated performance on perceptual line bisection (Armaghani et al., 2014; Cattaneo et al., 2014a; Hatin and Sykes Tottenham, 2016; Leggett et al., 2016). Two studies (Cattaneo et al., 2014a; Hatin and Sykes Tottenham, 2016) found that positive emotion (i.e., happy faces) induces a decrement of pseudoneglect (i.e., attenuates the leftward bias); one investigation reported mixed results (Leggett et al., 2016) and another (Armaghani et al., 2014) observed that both happy and sad faces enhanced the leftward bias compared to neutral faces.

This extreme variability in experimental results could be explained by considering individual differences. In fact, those very few studies that explored the effect of self-reported emotions and traits on pseudoneglect showed that positive affect (Drake and Myers, 2006) and positive attitude (Somma et al., 2021) are correlated with a rightward bias. Conversely, a recent study (Somma et al., 2021) investigating the impact of stressful situations (COVID-19 restrictions) on visuospatial attention found a positive correlation between the magnitude of pseudoneglect (probably due to greater activity in the right hemisphere) and perceived distress. This evidence suggests the need to gain a deeper understanding of the role of internal emotional states in the modulation of pseudoneglect. Specifically, since it has been found that higher levels of anxiety are related to hyperactivation of the right frontal and parietal cortex (Tomarken and Davidson, 1994; Blackhart et al., 2006; Mathersul et al., 2008), it may be that individual differences in trait anxiety can influence the magnitude of pseudoneglect phenomena. High-anxiety subjects may indeed exhibit a greater leftward bias in the bisection task (pseudoneglect) as a consequence of greater tonic activation of the right hemisphere.

Hence, the main aim of this study was to explore, for the first time, the relationship between trait anxiety and the pseudoneglect phenomenon. This topic may also be relevant to clarify previous research on visual attention bias. In fact, individual differences in emotional states, such as trait anxiety, may explain the inconsistency in the literature on pseudoneglect (Armaghani et al., 2014; Cattaneo et al., 2014a; Hatin and Sykes Tottenham, 2016; Leggett et al., 2016).

For our purpose, we assessed high- and low-anxiety participants using a line bisection task with emotional faces as flankers [the same used in Cattaneo et al. (2014a)]. Each test consisted of estimating the center of a line (of two different lengths and placed in different positions on the screen) flanked by two empty circles or containing a neutral, happy, or sad face.

## 2. Materials and methods

### 2.1. Participants

**Figure 1:**
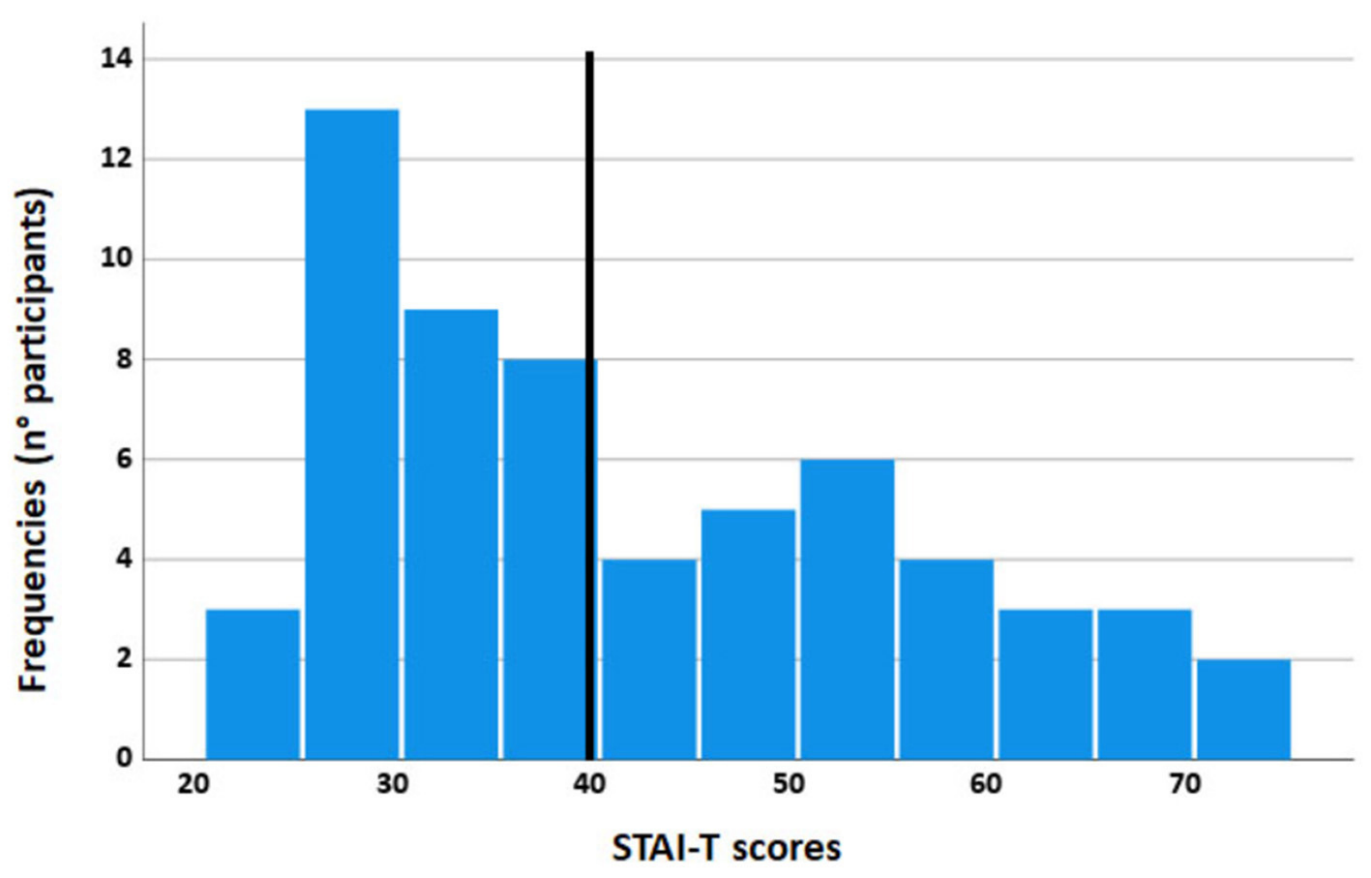
Frequency histogram of the *STAI-T* scores: the *y*-axis represents the number of participants, and the *x*-axis represents the *STAI-T* scores. The black vertical bar is the median.

A total of 60 volunteers (32 women, mean age = 24.60; SD = 3.01; age range: 18–31 years) were randomly selected from the psychology students' community of the University of Florence. The sample size was based on a Power (1 – beta) of 0.95 with an effect size of *f* = 0.38 (Cattaneo et al., 2014a) and alpha = 0.05. We computed (G^*^Power 3.1.9.7; Faul et al., 2007) a total sample size of at least 16 participants. We recruited a sample of 60 subjects, reaching a greater sensitivity up to f = 0.19. All participants were right-handed, had normal or corrected-to-normal vision, and had no history of mental illness. Participants were administered the Italian version of the subscale *STAI-T* of the *State Trait Anxiety Inventory* (*STAI*; Spielberger et al., 1970; Sanavio et al., 1997). Cronbach's alpha: 0.90. The *STAI-T* consists of a 20-item scale that measures relatively stable individual differences in anxiety propensity or differences in the predisposition to experience anxiety states. High-anxiety trait subjects were more susceptible to responding to situations perceived as threatening with significant increases in anxiety. A cut-off of 40 has been considered optimal to screen for the possible presence of anxiety disorders (Van Dam et al., 2013). We divided participants into high- and low-anxiety groups by the median split of the *STAI-T* score, which was 39, so that all the high-anxiety subjects had *STAI-T* scores ≥40 (see Figure 1).

Each group, the high-anxiety group (17 women, mean age = 24.40; SD = 3.31; age range: 18–30 years; mean *STAI-T* = 54.00; SE = 1.70) and the low-anxiety group (15 women, mean age = 24.80; SD = 2.66; age range: 21–30 years; mean *STAI T*= 30.36; SE = 0.79) was composed of 30 subjects. Two groups differed for *STAI-T* (*p* < 0.001) and did not differ for age (*p* = 0.613).

All participants gave their written informed consent to the procedure and the processing of personal data. The data were collected and processed anonymously. Prior to the evaluation, each subject was blind to the purpose of the study, which was carefully explained after the completion of the evaluation.

### 2.2. Face stimuli

The face set consisted of four different young Caucasian female identities and four male identities selected from the KDEF database (Lundqvist et al., 1998). For each face identity, we selected neutral, happy, and sad expressions (Figure 1) so that we could collect 24 facial stimuli (12 women and 12 men). We compared the value of arousal in the faces using a *t*-test (Lundqvist et al., 1998). This allowed us to check that (a) the happy and sad faces did not differ from each other for mean arousal (*p*_s_ > 0.05) and (b) both happy and sad faces differ from neutral faces for mean arousal (for both *p*_s_ < 0.05).

### 2.3. Bisection task

Every trial was characterized by the presence of a black line flanked by two circles (diameter of 2.5°, placed at a distance of seven pixels from the ends of the line).

The task included four experimental conditions: a “baseline condition” and three “emotional face conditions.” In the “baseline condition,” the circles were empty; in the “emotional face conditions,” the circles contained a face with a happy, sad, or neutral emotional expression.

To increase stimulus variability and reduce the possibility of estimating the center of the line by taking reference points on the frame of the screen, two different line lengths were used, measuring 8° (8 cm) and 12° (12 cm), that could appear in eight different positions: lines were always displaced 1° right or left from the center and displaced 1° or 3° up or down from the center. Thus, in each of the four experimental conditions, long and short lines appeared the same number of times in each of the eight possible positions.

We presented emotional expression in blocks; the decision was derived from the observation of the results reported by Schepman et al. (2012). In their experiment, the authors noticed a specific bias in a dichotic listening paradigm as a function of emotional valence that emerged only in the condition in which emotional stimuli were divided by blocks, and they found their results in accordance with Kinsbourne's hemispheric activation theory (1970), assuming that prolonged exposition to the same emotion would have been more able to promote the activation of the more specialized frontal areas depending on the value.

In the present experiment, for all participants, an experimental trial started from the baseline block, while the order of presentation of the other three blocks with emotional faces was randomized and counterbalanced across subjects. Before the experimental phase, a training phase was carried out to familiarize the participants with the task.

The baseline block consisted of 16 trials, eight for each line length, while the emotional faces block consisted of 32 trials. Each of the 24 facial stimuli appeared the same number of times at the left end and at the right end of the line. In each trial, the faces used as flankers belonged to the same gender, always expressing the same emotion. Each face was also presented in combination with itself and twice with other faces of the same gender (once as a left and once as a right flanker) and expressed the same emotion. For example, two faces flanked the short line in a trial, and in another trial of the same block, they appeared inverted at both ends of the long line.

**Figure 2:**
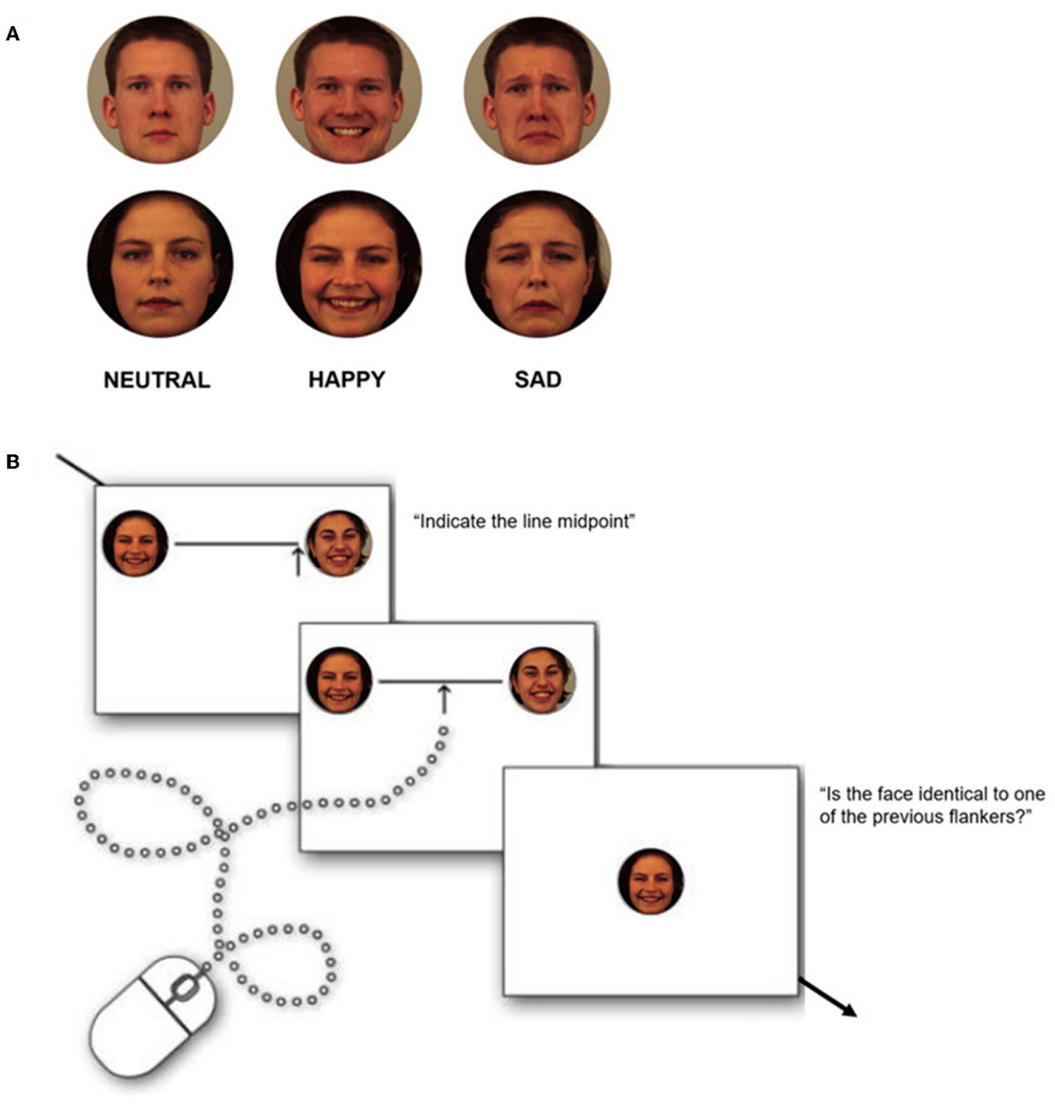
**(A)** Examples of face stimuli used as flankers in the line bisection task. **(B)** Illustration of the phases of the experimental test. Each line appeared randomly in eight different positions and was flanked by a face at both ends of the same kind and with the same emotional value. Each test followed a memory test. Facial images correspond to identity AF06, AF17, and BM11 from the KDEF open-access database, with permission from the ‘Psychology section, Department of Clinical Neuroscience, Karolinska Institute, Stockholm, Sweden’. Available online at: https://kdef.se/download-2/.

Participants were instructed to move the mouse pointer and select the midpoint of each line by clicking the left mouse button as accurately as possible. The mouse pointer was constrained to a vertical arrow and could be moved only in the horizontal plane, five pixels below the line. For each trial, the starting position of the mouse pointer was randomly assigned to the left or right end of the line on the screen. The line remained on the screen until a response was made. To make sure that each participant paid attention to the flankers, at the beginning of the emotional block, they were instructed to perform a memory test after every bisection test. Particularly after the line center had been estimated, the line disappeared and a single face appeared centrally. Participants had to indicate if the face was the same as one of the two that flanked the line in the bisection trial just conducted. They had to report their answer by pressing the left (yes) or right (no) mouse button, respectively. Refer to Figure 2 for the trial representation. The faces presented in the memory test always belonged to the same gender and showed the same emotion as the faces used as flankers in the previous trial. Furthermore, for half of the trials, they were the same as one of the flankers used in the bisection task (half of the time the same as the one on the left, the other half to the right). Task execution was performed in approximately 17/20 min.

### 2.4. Procedure

Participants underwent a computerized version of the line bisection task using the face stimuli as flankers (Cattaneo et al., 2014a). The open-source software OpenSesame 3.2.6 Kafkaesque Koffka (Mathôt et al., 2012) was used for stimuli production and response recording. Participants were seated in front of a 15.6-inch laptop screen (1,366 × 768 pixels) at an approximate distance of 57 cm. After the task, participants filled in the *STAI-T*. The experiment required approximately half an hour.

## 3. Results

To confirm previous studies that employed the bisection task (Cattaneo et al., 2014a,b), we first analyzed the global performance of the 60 participants (not considering the differences in trait anxiety scores).

### 3.1. Accuracy on the memory task of all subjects

As it has been done in previous studies (Cattaneo et al., 2012, 2014a,b), to verify that participants paid attention to the faces that flanked the lines, we analyzed the accuracy of the recognition memory task with a repeated measure ANOVA with two levels of factor Side (left vs. right) and three levels of factor condition (neutral, sad, and happy faces). No main effect or interaction was significant (all *p*_s_ > 0.05*)*. However, accuracy was high in all the conditions (neutral = 0.940, SD = 0.103; sad = 0.936, SD = 0.109; happy = 0.945, SD = 0.104), indicating that the participants did pay attention to the faces.

### 3.2. Bisection task performance of all the subjects

Responses were quantified as deviations from the veridical line midpoint by means of signed percentage scores (Cattaneo et al., 2014a). Specifically, the bisection bias was quantified as the difference between the chosen midpoint (calculated from the left extremity of the line) and the true half-length. Both measures were estimated in pixels. The sign of this score could be either positive or negative, reflecting a mid-point estimation on the right or left side of the true line center, respectively. Finally, this value was divided by the true half-length and multiplied by 100. This score represents, therefore, a proportion of the bisection bias in online length. As such, it does not allow the investigation of the effects of line length differences *per se*, as the proportion rules out information about the original line size. Therefore, the score standardizes the bias effect among different lengths.

First, we checked that the data were normally distributed using the Kolmogorov-Smirnov test. The results showed normal distributions for all conditions (Baseline, Neutral, Sad, and Happy; all *p*_s_ > 0.05). To verify the presence of pseudoneglect in our participants, we performed a series of one-sample *t*-tests (*t*-tests against 0) on the mean bisection bias of each participant, separately on all conditions (Baseline, Neutral, Sad, and Happy). The results showed that the pseudoneglect was present for the following conditions: Baseline: mean (SE ± 0.29) = −0.80, *t* = −2.77, *p* = 0.004; Neutral: mean (SE ± 0.25) = −0.60, *t* = −2.36, *p* = 0.011; and Sad: mean (SE ±0.26) = −0.72, *t* = −2.82, *p* = 0.003, but not for the condition Happy: mean (SE ± 0.25) = −0.09; *t* = −0.49, *p* = 0.314.

**Figure 3:**
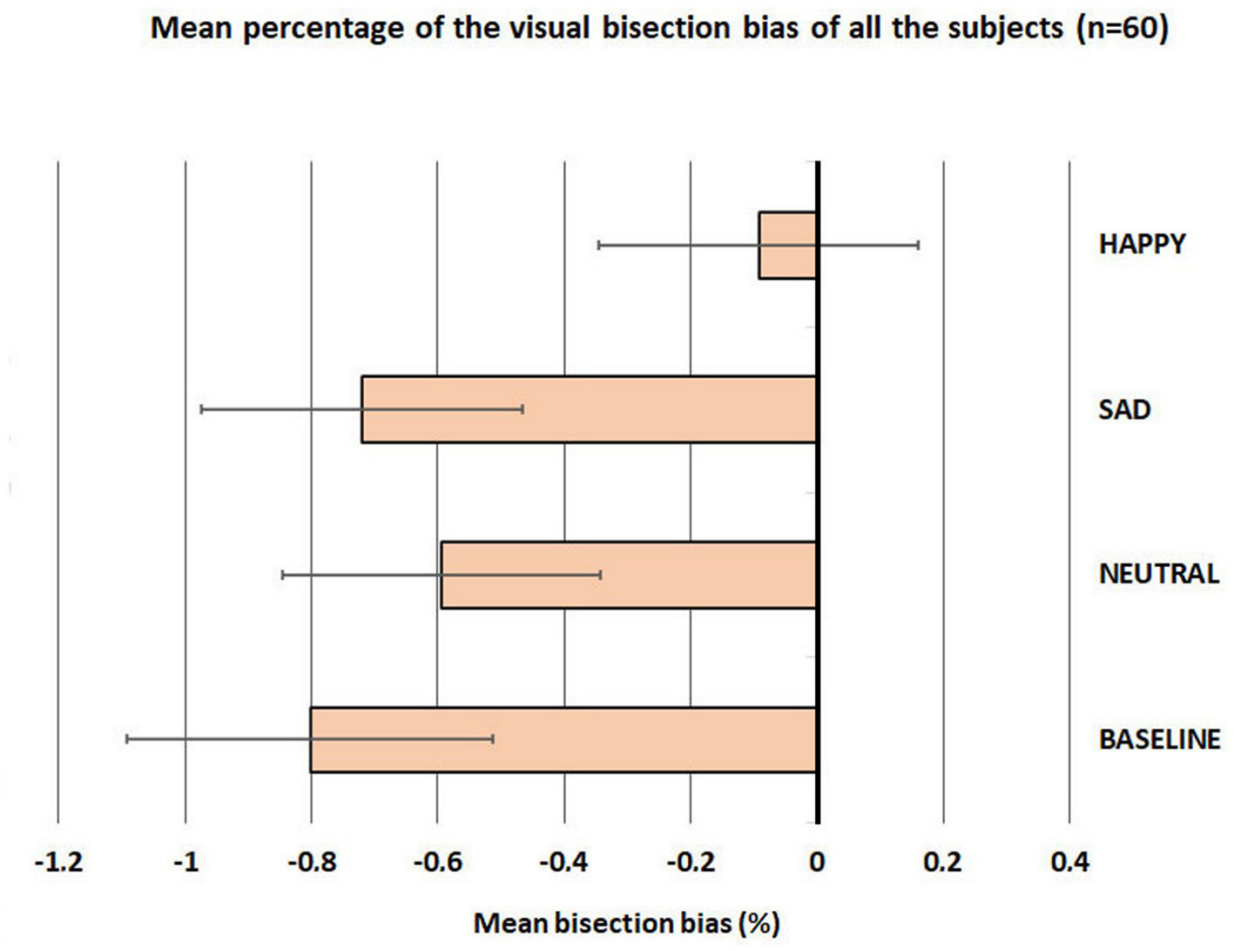
Mean percentage (±standard error) of the visual bisection bias of all subjects (60 participants).

The one-sample *t*-tests were complemented by a repeated-measure ANOVA with four levels of the factor condition (Baseline, Neutral, Sad, and Happy); this analysis was conducted on the mean bisection bias of each participant. To correct the mean (SE ±0.29) = −0.80, violations of the sphericity assumption, and inherent in repeated-measure designs, the Greenhouse–Geisser correction was applied, and the adjusted degrees of freedom rounded to the nearest whole number are reported. The results evidenced a significant main effect of condition: *F*_(3, 160)_ = 2.863; *p* = 0.043, $\eta_{\text{p}}^{2}$ = 0.705. *Post-hoc t*-test showed that happy faces (mean = −0.09; SE = 0.25) reduced the pseudoneglect (they shifted the bisection bias significantly to the right) compared to Baseline (mean = −0.80; SE = 0.29; *p* = 0.008), neutral faces (mean = −0.60; SE = 0.25; *p* = 0.044), and sad faces (mean = −0.72; SE = 0.26; *p* = 0.005; see Figure 3).

In a similar way to what was done to analyze the performances of all subjects, we examined prior recognition memory and then the bisection task performance as a function of trait anxiety.

### 3.3. Accuracy on memory task as a function of trait anxiety

A repeated measure ANOVA with two levels of the factor side (left vs. right), three levels of the factor condition (neutral, sad, and happy faces), and two levels of groups (high-anxiety vs. low-anxiety) as a between-subjects factor was conducted on the accuracy scores of each subject. No main effect or interaction reached significance (all *p*_s_ > 0.05).

### 3.4. Bisection task as a function of trait anxiety

To verify the relationship between trait anxiety and the pseudoneglect phenomenon, we performed a correlation analysis between the *STAI-T* scores and the mean bisection bias of participants for the four conditions (Baseline, Neutral, Sad, and Happy). The results showed a significant correlation for the following conditions: Neutral (*r* = −0.44; *p* = 0.001), Sad (*r* = −0.41; *p* = 0.001), and Happy (*r* = −0.35; *p* = 0.007).

To investigate this association between the trait anxiety and the mean bisection performance more systematically, we performed a series of sample *t*-tests (*t*-tests against 0) within each group (high-anxiety and low-anxiety separately) on the conditions (Baseline, Neutral, Sad, and Happy). In the low-anxiety group, the results showed that the pseudoneglect was present only for the condition (Baseline: *t* = −1.68, *p* = 0.05), but not for the other conditions (Neutral: *t* = 0.06, *p* = 0.48; Sad: *t* = 0.47, *p* = 0.32; and Happy: *t* = 1.13, *p* = 0.13). In the high-anxiety group, the results showed that the pseudoneglect was present in all the following conditions: Baseline: *t* = −2.23, *p* = 0.017; Neutral: *t* = −3.36, *p* = 0.001; Sad: *t* = −5.34, *p* ≤ 0.001; and Happy: *t* = −2.13, *p* = 0.021. For the low-anxiety participants, the mean values were as follows: Baseline = −0.66, SE = 0.4; Neutral = 0.02, SE = 0.32; Sad = 0.16, SE = 0.35; and Happy = 0.43, SE = 0.38. For the high-anxiety participants, the mean values were as follows: Baseline = −0.95, SE = 0.43; Neutral = −1.21, SE = 0.36; Sad = −1.60, SE = 0.30; and Happy = −0.68, SE = 0.32.

To compare the performance between the groups, we performed a repeated-measure ANOVA with two levels of groups (high-anxiety vs. low-anxiety) as a between-subjects factor and four levels of condition (Baseline, Neutral, Sad, and Happy) as a within-subjects factor. This analysis evidenced the main effect of the groups, *F*_(1, 58)_ =7.350; *p* = 0.009, $\eta_{\text{p}}^{2}$ = 0.112 (high-anxiety: mean = −1.111, SE = 0.29; low-anxiety: mean = −0.010, SE = 0.28), indicating that pseudoneglect (leftward bisection error) was greater in the high-anxiety subjects compared to the low-anxiety subjects. Moreover, the significant main effect of condition, *F*_(3, 160)_ =2.960; *p* = 0.038, $\eta_{\text{p}}^{2}$ = 0.049, showed that pseudoneglect was greater when the lines were flanked by sad faces (mean = −0.72; SE = 0.26); compared with happy faces (mean = −0.09; SE = 0.25; *p* = 0.044).

**Figure 4:**
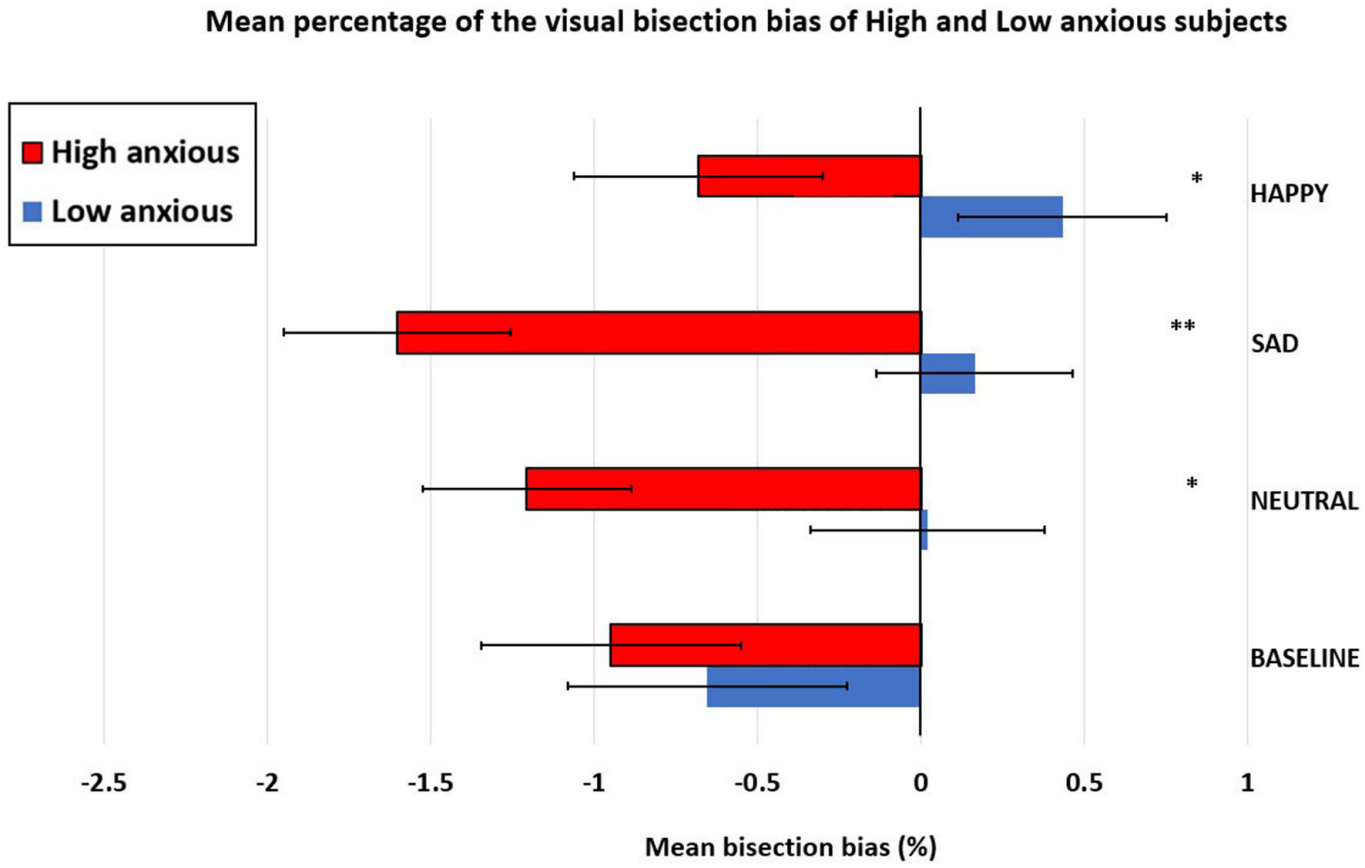
Mean percentage (±standard error) of the visual bisection bias in low- and high-anxious participants (between group differences: *p < 0.05; **p < 0.01).

Furthermore, the significant interaction condition × group, *F*_(3, 160)_ = 2.997; *p* = 0.036, $\eta_{\text{p}}^{2}$ = 0.049 emerged. *Post-hoc* comparison, computed with Bonferroni's correction, showed that the high-anxiety participants had greater pseudoneglect compared to the low-anxiety participants for neutral (high-anxiety: mean = −1.21, SE = 0.36; low-anxiety: mean = 0.02, SE = 0.32; *p* = 0.013), sad (high-anxiety: mean = −1.60, SE = 0.30; low-anxiety: mean = 0.16, SE = 0.35; *p* = 0.001), and happy faces (high-anxiety: mean = −0.68, SE = 0.32; low-anxiety: mean = 0.43, SE = 0.38; *p* = 0.029). No difference between groups in the bisection performance was evidenced for the lines flanked by empty circles (Baseline; high-anxiety: mean = −0.95, SE = 0.43; low-anxiety: mean = −0.66, SE = 0.4; *p* = 0.615). In addition, the *post-hoc* comparison computed within groups evidenced that in the high-anxiety group, pseudoneglect was significantly reduced (reduction of leftward shift) for happy faces (mean = −0.68, SE = 0.32) compared to sad faces (mean = −1.60, SE = 0.30, *p* = 0.021). Differently, in the low-anxiety group, pseudoneglect was significantly reduced for happy (mean = 0.43, SE = 0.38) compared to Baseline (mean = −0.66, SE = 0.4; *p* = 0.027; see Figure 4).

## 4. Discussion

The present study was conducted with the aim of shedding light on the role of emotion in modulating pseudoneglect in view of the mixed results of the few previous studies that investigated how the presentation of emotional faces with positive and negative valence moderated performance on the line bisection task (Armaghani et al., 2014; Cattaneo et al., 2014a; Hatin and Sykes Tottenham, 2016; Leggett et al., 2016). An additional objective was to explore whether and to what extent trait anxiety may influence the pseudoneglect phenomenon. The hypothesis that anxiety or stress may modulate the extent of attentional bias in bisection line performance has been scarcely investigated (Drake and Myers, 2006; Somma et al., 2021), despite the evidence that negative self-reported affect can be associated with hyperactivation of the right frontal and parietal cortex (Davidson and Hugdahl, 1995; Blackhart et al., 2006; Mathersul et al., 2008).

When we take into account the performance of all participants in the current investigation, our results are consistent with two previous studies (Cattaneo et al., 2014a; Hatin and Sykes Tottenham, 2016), showing that happy faces decrease pseudoneglect, reducing the leftward bisection error when compared to Baseline (empty circle flanked lines), as well as to neutral and negative (sad faces) stimuli. The extent of the pseudoneglect in our results is substantially comparable to previous studies (Cattaneo et al., 2014a,b; Hatin and Sykes Tottenham, 2016). The neurobiological underpinnings of the leftward bias (pseudoneglect) are likely to depend on hemispheric asymmetries in attentional frontoparietal networks, which are modulated by emotional processing (de Schotten et al., 2005; Corbetta et al., 2008; Bartolomeo and Malkinson, 2019). Specifically, the finding that happy faces enhance the allocation of spatial attention toward the right hemispace is consistent with the “valence-specific hypothesis” (Reuter-Lorenz et al., 1983; Davidson, 1992, 1998), which postulates that positive emotions preferentially activate the left hemisphere, whereas negative emotions would mainly be processed by the right hemisphere.

Further evidence of a different involvement of the hemispheres in positive vs. negative emotional states (“valence-specific hypothesis”: Reuter-Lorenz et al., 1983; Davidson, 1992, 1998) also emerges when we take into account the impact of trait anxiety on the pseudoneglect phenomenon. Remarkably, although we evaluated a non-clinical population, the high-anxiety individuals we identified by the median split of the *STAI-T* values at a score of 39 can be considered a population with the possible presence of anxiety disorders (Van Dam et al., 2013; Balsamo and Carlucci, 2020).

Our high-anxiety participants showed a significantly greater leftward bisection shift when compared to low-anxiety subjects in all the conditions, excluding Baseline. Indeed, in the baseline condition, both groups did not differ in showing the leftward bisection error. This finding is in line with most studies reporting the pseudoneglect phenomenon (Jewell and McCourt, 2000; Friederich et al., 2018). Thus, results clearly indicate that between groups, observed differences were specifically driven by the faces. This possibility is supported by an extensive literature review that investigated the effect of emotions on visual attention as a function of anxiety (Bradley et al., 1998; Pishyar et al., 2008). Indeed, several studies found a modulation of vigilance toward faces, and especially toward emotional negative expression, in high- and low-anxiety individuals (Bar-Haim et al., 2007; Frewen et al., 2008; Schulz et al., 2013). Faces and facial expressions may be especially important for anxious individuals since this information may inform them about threats, negative situations, and/or evaluation by others (Rapee and Heimberg, 1997). This is also confirmed by neurophysiological studies that examined trait anxiety-related attentional bias (Fox et al., 2008; Eldar et al., 2010), showing that very early differences in the processing of faces and emotional faces between high- and low-anxiety subjects may be due to enhanced attentional processing in the visual cortex, which correlates with increased amygdala activity (Carlson et al., 2009).

Consistent with this literature, we found that high-anxiety participants allocated visual attention differently from low-anxiety participants, exhibiting greater pseudoneglect in all the conditions in which bisection lines were flanked by faces. Furthermore, *post-hoc* comparison within our groups showed that, although the pseudoneglect persists in high-anxiety participants, it decreases (smaller leftward bisection shift) for happy faces compared to the other face expressions (both sad and neutral). Conversely, in the low-anxiety group, there was no pseudoneglect in any of the face conditions (happy, sad, and neutral), which did not differ from each other.

Based on the results obtained, it can be hypothesized that the high-anxiety trait increases pseudoneglect through enhanced right hemispheric activity. Conversely, a low level of anxiety, corresponding to positive emotional status and wellbeing, can reduce the pseudoneglect by increasing the left hemispheric activation (and hence reducing or eliminating the leftward bisection shift). Our hypothesis agrees with previous evidence of a predominant right hemisphere activity during negative and stressful situations (Compton et al., 2000; Ocklenburg et al., 2016; Bartolomeo and Malkinson, 2019; Somma et al., 2021). Several studies found, in fact, that acute and chronic distress can thus influence lateralized behavior in humans and animals as a result of higher right-hemisphere activation (Ocklenburg et al., 2016). A recent study (Somma et al., 2021), which investigated the impact of stressful situations (COVID-19 restrictions) on visuospatial attention, found that the pseudoneglect increment (hence, greater activation of the right hemisphere) was positively correlated with perceived distress and negatively correlated with a positive attitude. While right hemispheric asymmetry seems involved in distress conditions such as high anxiety, resiliency (Kong et al., 2018), and positive emotional states (Somma et al., 2021) seem to be associated mainly with left hemispheric activity. As reported by Santarnecchi and colleagues (2018), positive coping strategies would indeed relate to greater left hemisphere activation and connectivity, particularly concerning the left angular gyrus. The neural mechanisms underlying these emotion-related asymmetries are, however, still under debate. In fact, some studies (Soares et al., 2013; Santarnecchi et al., 2018; Brem et al., 2020) suggest that distress may produce, over time, an increment in the connectivity in the right hemisphere attentional networks (and hence hyperactivation of the right hemisphere). Differently, other studies (Eden et al., 2015) that found a reduction in the connectivity between the amygdala and ventro-medial prefrontal cortex of the right hemisphere in persons with high-anxiety traits attribute the anxiety-related hyperactivation of the right hemisphere to failure in modulation and suppression of the activity of the right amygdala (and hence hyperstimulation of the right prefrontal cortex).

Regardless of the neural underpinnings, the anxiety-related hyperactivation of the right hemisphere may explain why our high-anxiety participants showed greater pseudoneglect (more leftward shift) in all the line bisection conditions with faces (when compared to low-anxiety participants) and also why there was no reduction of pseudoneglect for happy faces. Some evidence (Eden et al., 2015) showed stronger connectivity between the amygdala and ventro-medial right prefrontal cortex in people with low-anxiety traits. It may be that our low-anxiety group did not show pseudoneglect for face conditions (happy, sad, and neutral) because they are able to both better suppress the activity of the right amygdala and activate emotion regulation and re-appraisal, which are primarily associated with left-hemispheric processing in these structures (Kim and Bell, 2006; Kim et al., 2012).

All in all, by highlighting the difference in the pattern of allocation of visual attention in low- and high-anxiety subjects (likely due to the different engagement of the two hemispheres in trait anxiety), the present study contributes to clarifying the variability in the results of previous studies on pseudoneglect (Armaghani et al., 2014; Cattaneo et al., 2014a; Hatin and Sykes Tottenham, 2016; Leggett et al., 2016). In fact, considerable discrepancies exist in the literature reporting how the presentation of emotional faces with positive and negative valence modulated the performance on the perceptual line bisection, which may also be due to individual differences in the anxiety levels of the participants. In this vein, our work contributes to a better understanding of the spatial attentional bias as it stresses the relevance of considering emotional individual differences in studying the pseudoneglect phenomenon.

## Data availability statement

The raw data supporting the conclusions of this article will be made available by the authors, upon reasonable request to interested researchers.

## Ethics statement

The studies involving human participants were reviewed and approved by Commissione Etica per la Ricerca - University of Florence. The patients/participants provided their written informed consent to participate in this study.

## Author contributions

Conceptualization: MPV, SR, MTT, and FG. Methodology: GR, VB, and SR. Formal analysis: VB, MTT, and GR. Writing—original draft preparation: MPV, FG, and SR. Writing—review and editing: MPV and SR. All authors have read and agreed to the published version of this manuscript.
